# Taking Cold

**Published:** 1881-12

**Authors:** 


### TAKING COLD.

IT IS impossible to adapt ourselves to sudden changes of the weather so as at all times to escape its dangers; but it is an easy matter to establish certain rules for our guidance, when exposed to these dangers, the observance of which will generally secure our safety. The instinct of self-protection is common to all living things; thus, when men or animals are left to themselves they naturally avoid dangers that threaten them, whether of heat or cold, hunger or thirst. But many of the customs and habits of society, the artificial methods of living, and the prevailing ignorance of sanitary laws, condemn a large percentage of the human race to almost perpetual invalidity. Let those who may doubt this assertion look about them, and see how many persons of their acquaintance remain perfectly well during three consecutive months. A majority of diseases come from taking cold, and all diseases are aggravated by cold. To avoid taking cold, then, is generally to avoid disease. The ways in which people take cold are so numerous and so insidious that it would not be possible to enumerate them. I will only mention some of the more common, and suggest a few simple precautionary measures, which, if generally adopted as rules, would prolong many valuable lives.

Suppose we visit the largest factory in the world; how we wonder at the genius that set those hundreds of looms and hundreds of thousands of spindles in motion; yet multiply them all by ten thousand, and they will not equal in number the instruments and devices that constitute life, and the simplest of which is a profound mystery in comparison to the grandest achievement of human knowledge and human skill.

It seems impossible that any one should long remain in health, when that condition can only be maintained by a perfect and harmonious working of life's machinery. But although nature tolerates a great many abuses for a season, if these are persisted in she rebels. Whatever pleasures there are in life are most enjoyed by those of soundest health.

In order to enjoy good health it is not necessary to watch the operations of the system; in fact, that is one of the worst things one can do. Be nervously anxious about the heart, and watch its beatings, and you will be lucky if you escape at least a functional derangement of that organ; and so with the kidneys and other important parts. But if we supply the stomach with a proper amount of wholesome food, avoid all things that are pernicious, and protect the body from sudden extremes of heat and cold, we can safely leave the mysterious operations that we call life to be wrought out in nature's laboratory. Of these we know but little. We know that when food enters the stomach it passes through a process by which most of the nutritive portion is separated and taken up by the absorbents and carried into the circulation. After undergoing other changes, the fluid portions are passed out of the body through the kidneys and the skin, glands and mucous membranes. Now, if the equilibrium is not maintained; that is, if the skin and the kidneys can not, for any reason, conduct these effete and poisonous matters from the system as rapidly as they accumulate, the health suffers just in proportion to the quantity of this poisonous matter that is retained. Were none to escape, death would result in an hour.

To suddenly check perspiration is to check the elimination of poison from the system. It is almost always caused by cold, which closes up the pores of the skin at the part affected. A cold in the head checks the flow into the nasal passages. A draught of air on the back often checks excretion at that point, and the matter is thrown back to the lungs, producing congestion or inflammation. If you are where you can not avoid a draught of air turn the face to it. If you are compelled to sleep on the ground, sleep with the face down. All the vital organs are attached to the inside of the back and cold easily strikes through from the outside, whereas they are better protected in front. People seldom take cold who walk or ride in the open air in every kind of weather, if the feet are kept warm and dry and the body, particularly the back, is properly protected with clothing. If a cold is taken under these conditions it seldom amounts to anything serious. But a cold taken on coming out of a crowded and poorly-ventilated room or railroad car is likely to result in pneumonia or typhoid fever, the reason being that a vitiated atmosphere impairs the power to resist disease.

There are two remedies against this evil. If one finds himself in a crowded and badly-ventilated room the best plan is to leave as promptly as possible. If this is not expedient he should have on going out some garment that will completely protect him.

---

TEMPERANCE and prosperity go hand in hand.

